# Malignant Struma ovarii in a 30-year old nulliparous patient

**DOI:** 10.1186/s13044-017-0038-1

**Published:** 2017-05-30

**Authors:** J. Colin Boyd, Blair A. Williams, Matthew H. Rigby, Katharina Kieser, Saul Offman, Hemlata Shirsat, Jonathan R. B. Trites, S. Mark Taylor, Robert D. Hart

**Affiliations:** 1 Dalhousie Medical School, Dalhousie University, 5849 University Avenue, Halifax, NS B3H 4H7 Canada; 2 Division of Otolaryngology – Head and Neck Surgery, Department of Surgery, Dalhousie University, Halifax, NS Canada; 3 Department of Obstetrics and Gynecology, Dalhousie University, Halifax, NS Canada; 4 Department of Pathology, Dalhousie University, Halifax, NS Canada

**Keywords:** Thyroid carcinoma, Thyroidectomy, I-131, Radioiodine, Oncology

## Abstract

**Background:**

Struma ovarii is a rare monodermal germ cell tumor where the ovary is comprised of at least half thyroid tissue. This phenomenon may indicate an embryological origin.

**Case presentation:**

A 30-year old nulliparous woman presented with acute right lower quadrant pain and underwent laparoscopic right salpingo-oophorectomy. The excised ovarian mass showed evidence of struma-derived papillary thyroid carcinoma. Ultrasound of the thyroid showed mild enlargement with two solid nodules. A fine needle aspirate of a thyroid nodule was positive for malignancy and a total thyroidectomy was performed. Microscopic features of the thyroid were consistent with papillary thyroid carcinoma. The two tumours were considered as synchronous independent primaries based on their histological presentation.

**Conclusions:**

We believe that aggressive surgical management followed by radioiodine therapy is best to reduce recurrence risk and optimize survival. The broad scope of interventions needed to treat malignant struma ovarii require a strong interdisciplinary team.

## Background

Struma ovarii is a monodermal germ cell tumor first described by R. Boëttlin in 1889 [1]. It represents 2–3% of all ovarian tumours and by definition must be comprised of at least half thyroid tissue [2–4]. Struma ovarii is rare; less than 200 cases have been reported in the medical literature [3, 5]. Malignant struma ovarii is rarer still, with malignancy occurring in less than 5% of cases and metastases highly uncommon [6, 7]. However, recurrence rates vary widely and have been reported from 7.5–35% [4, 6, 8]. Goffredo et al. [3] report excellent survival rates for patients with malignant struma ovarii but emphasize the high risk of aggressive thyroid cancers, which occurred in 6 of their 68 patients.

Multifocal thyroid-type tumors that arise synchronously in separate locations may represent a common genetic predisposition for tumor formation, likely occurring in embryogenesis. These cells differentiate into thyroid-type tissue and, after exposure to carcinogens later in life, undergo distinct but parallel tumorigenesis [9]. This phenomenon may similarly be explained by “field cancerization”, where exposure to carcinogens creates a carcinogen-damaged field of thyroid type tissue regardless of anatomic location [10].

Given its rarity, little is known about malignant struma ovarii and accordingly there are few guidelines to manage its treatment [4, 8, 9, 11, 12]. A significant issue for clinicians to consider is the intensity of treatment. This may include pelvic surgery, thyroidectomy and radioactive iodine (I-131), which must be balanced against the risks of infertility and the low rate of metastasis.

Here we present the case of a 30 year-old nulliparous patient with malignant struma ovarii and concomitant papillary thyroid carcinoma.

## Case Presentation

A 30-year old nulliparous woman presented to her regional hospital with acute right lower quadrant pain. She was otherwise healthy with no significant past medical history. She reported smoking 10–15 cigarettes per day. A pelvic mass was palpated posteriorly and a pelvic ultrasound revealed a right adnexal mass suggestive of an ovarian cyst, with the possibility of torsion due to the patient’s level of discomfort. The patient was taken to the operating room and underwent a laparoscopic right salpingo-oophorectomy.

**Fig. 1:**
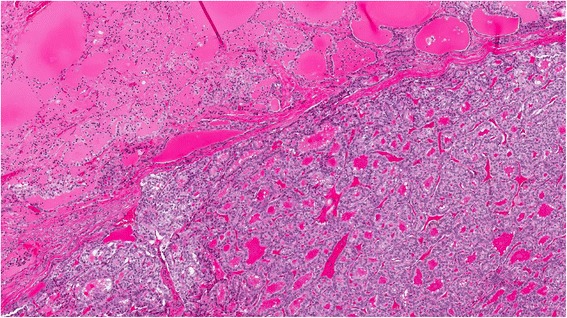
Struma ovarii with papillary thyroid carcinoma (H&E 4X)

**Fig. 2:**
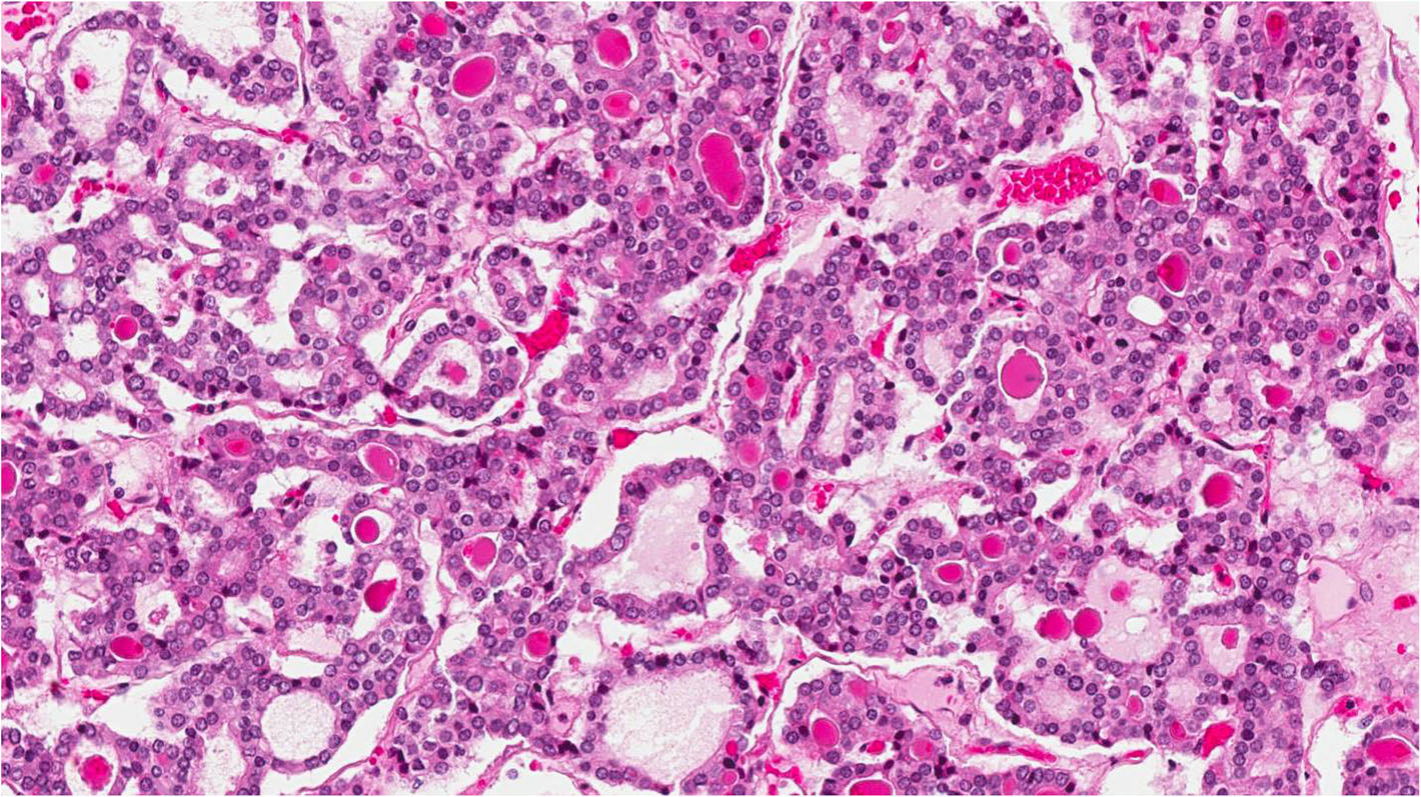
Papillary thyroid carcinoma in ovary (H&E 20X)

The ovarian mass (8.5 x 5.0 x 4.0 cm) was solid and cystic, with cysts containing milky-mucoid material. The solid area had a characteristic red-brown cut surface and a tooth was also identified. Histologic sections showed struma ovarii (Fig. 1) with limited non-strumal teratomatous elements (i.e. epidermal derivatives). The thyroid-type tissue showed multiple zones of increased cellularity, crowding, and nuclear alteration (enlargement, clearing, grooves, and membrane irregularity) along with scattered mitotic figures (Fig. 2). The features were diagnostic of struma-derived papillary thyroid carcinoma. Positive immunohistochemical staining for HBME1, Galectin 3 and CK19 supported the histologic impression. Neither surface involvement nor lymphovascular space invasion was identified.

The patient was referred to the Gynecology Oncology Cancer Site Team at a tertiary care centre who also involved the care of a thyroid team including Otolaryngology and Radiation Oncology.

Workup for thyroglobulin, serum TSH, anti-thyroglobulin antibody, and free T4 were performed and were consistent with euthyroid status. Ultrasound of the thyroid showed a mildly enlarged gland with a 1.1 cm solid isoechoic nodule in the upper pole of the right thyroid gland and a 5 mm hypoechoic solid nodule in the interpolar region of the left thyroid gland. Colloid cysts bilaterally measured up to 1.7 cm. An 18 F-FDG PET scan was conducted from skull base to proximal thighs and identified two low-density lesions in the right lobe of the thyroid with otherwise unremarkable uptake throughout the thyroid gland. However, absence of uptake FDG on PET does not exclude thyroid malignancy as most are low-grade differentiated thyroid cancers which are not particularly FDG-avid.

The combined results of the ultrasound and PET scan prompted referral for an ultrasound guided aspiration biopsy. Cytology results of the fine needle aspiration on right lobe nodule of thyroid were positive for malignancy, consistent with papillary thyroid carcinoma. These findings were discussed with the patient and a recommendation of total thyroidectomy was made. The thyroid was resected without complication.

The thyroid showed multiple cysts in both lobes and a solid 1.0 cm lesion in the lower pole of the right lobe. The microscopic features were consistent with papillary thyroid carcinoma. Neither lymphovascular space invasion nor extra thyroidal extension was identified.

The two tumours were interpreted as synchronous independent primaries. The presence of admixed epidermal derivatives in the ovary reflected primary teratomatous origin. The thyroid carcinoma was likewise deemed to be a primary, due to the distinct histomorphology, lack of aggressive features, absence of lymphatic or capsular/surface invasion in either organ, and absence of metastases elsewhere.

Following thyroidectomy, the patient was seen by Radiation Oncology who recommended I-131 therapy. She was treated on an outpatient basis and received a 100 mCi dose of I-131. The procedure was tolerated well and there were no complications. The follow-up whole body I-131 scan detected no evidence of nodal or distant metastatic disease.

## Discussion and Conclusions

Struma ovarii is a rare ovarian tumor, with malignancy reported in less than 5% of cases [3, 6]. Metastases are uncommon, occurring in 5–23%, of cases and mostly contained within the abdomen [12, 13]. Long-term follow up is crucial, as recurrence rates range from 7.5 to 35% [4, 6, 8]. Fortunately, survival rates after malignant struma ovarii are high: 92–96.7% at 5-years, 85–94.3% at 10-years, 84.9% at 20-years, and 79% at 25 yrs [3, 14]. To date, there has been no evidence of metastasis in the current case and close follow-up will continue to ensure early detection of any potential recurrence.

The rarity of malignant struma ovarii has limited our understanding of the natural course of the disease and as such the best approach to treatment is unknown [9, 15]. Various treatment strategies have been suggested, but no diagnostic or treatment guidelines have been established [3, 4, 11].

An important consideration in treatment of struma ovarii, given its occurrence in a reproductive organ and often in a younger population, is the threat to fertility. While the most comprehensive treatment for malignant struma ovarii would be abdominal hysterectomy and bilateral salpingo-oophorectomy with omentectomy, some have cautioned against overtreatment given the significant implication of permanent infertility [3, 8, 11]. Gynecologic intervention in the case presented above was limited to a unilateral oophorectomy to preserve fertility [6, 11] in our 30 year-old nulliparous patient. A prophylactic contralateral procedure may be considered in the future should the preservation of fertility become unnecessary.

A particularly contentious issue in the treatment of struma ovarii has been at what point to consider the use of thyroidectomy and radioactive iodine ablation therapy. Some conservative approaches suggest that, in the absence of high-risk features, surgical resection of the ovarian tumor may be adequate [9] and that thyroidectomy and radioiodine therapy should be reserved for metastases or recurrent disease [14]. More aggressive recommendations have suggested thyroidectomy and ablation therapy for malignant struma ovarii tumors with the following characteristics: ≥ 1 cm [15], disease outside the ovary, or with aggressive histological features [8]. Further to that, Marcy et al. [5] have suggested thyroidectomy be mandatory and performed quickly following pelvic surgery to preclude potentially lethal metastatic outcomes.

Generally, thyroidectomy and radioablation iodine therapy appear to be well-accepted practices for malignant struma ovarii in the literature [6, 9]. Importantly, there is some evidence to suggest that aggressive therapy including radioablation may provide better long term outcomes in cases of malignant struma ovarii. DeSimone et al. [6] reviewed 24 cases of malignant struma ovarii and found those who had thyroidectomy and radioactive ablation had no recurrence of disease compared to 50% recurrence in those who had surgery alone. Management of the current case seems to be in keeping with previous cases [4, 9, 15]. We believe that care of our patient is greatly improved by involving an interdisciplinary team including Gynecologic-Oncology, Radiation-Oncology, Endocrinology, Pathology, and Otolaryngology-Head and Neck Surgery.

The phenomenon of thyroid cancer concurrent with malignant struma ovarii, as described in the current case, has been observed previously [3, 9, 16]. Leong et al. [9] hypothesized that early genomic instability may explain tumorgenesis in discrete sites of thyroid-type tissue and explain the different tissue histology between thyroid and ovary tumours. Similarly, it has been hypothesized that mutations in the BRAF gene may be responsible for the mutual pathogenesis for papillary thyroid cancers at distinct locations in the body [16]. Six of the ten individuals with a cancer diagnosis secondary to malignant struma ovarii were thyroid cancers as reported by Goffredo et al. [3]. The authors indicate that this is an above average proportion of thyroid malignancies compared to the general public and that these patients had more invasive disease than would typically be expected [3]. Taken together, it appears that malignant struma ovarii poses a significant risk of additional thyroid cancer and clinicians would be wise to investigate that possibility at their earliest opportunity.

In conclusion, though no guideline recommendations exist for management of malignant struma ovarii, a generally accepted approach involving thyroidectomy and radioactive iodine ablation is emerging. Based on our findings and reports in the literature we believe that aggressive surgical management followed by adjuvant radioiodine therapy is best to reduce recurrence risk and optimize survival. Gynecologic intervention was done cautiously to protect fertility in this young female patient. Physicians should be aware of the relationship between malignant struma ovarii and potential concomitant malignant disease in the thyroid.

## Abbreviations

CK19: Cytokeratin 19
FDG: Fluorodeoxyglucose
mCi: Millicurie
PET: Positron emission tomography
TSH: Thyroid stimulating hormone
